# Nrf2 Plays a Protective Role Against Intravascular Hemolysis-Mediated Acute Kidney Injury

**DOI:** 10.3389/fphar.2019.00740

**Published:** 2019-07-03

**Authors:** Alfonso Rubio-Navarro, Cristina Vázquez-Carballo, Melania Guerrero-Hue, Cristina García-Caballero, Carmen Herencia, Eduardo Gutiérrez, Claudia Yuste, Ángel Sevillano, Manuel Praga, Javier Egea, Pablo Cannata, Isabel Cortegano, Belén de Andrés, María Luisa Gaspar, Susana Cadenas, Patrycja Michalska, Rafael León, Alberto Ortiz, Jesús Egido, Juan Antonio Moreno

**Affiliations:** ^1^Renal, Vascular and Diabetes Research Lab, Instituto de Investigación Sanitaria-Fundación Jiménez Díaz, Autónoma University, Madrid, Spain; ^2^Department of Nephrology, Hospital 12 de Octubre, Madrid, Spain; ^3^Instituto de Investigación Sanitaria-Hospital Universitario de la Princesa, Madrid, Spain; ^4^Instituto Teófilo Hernando, Departamento de Farmacología y Terapéutica, Facultad de Medicina, UAM, Madrid, Spain; ^5^Hospital Santa Cristina, Madrid, Spain; ^6^Pathology Department, Fundación Instituto de Investigaciones Sanitarias-Fundación Jiménez Díaz, Autónoma University, Madrid, Spain; ^7^Immunology Department, Centro Nacional de Microbiologìa, Instituto de Salud Carlos III (ISCIII), Madrid, Spain; ^8^Centro de Biología Molecular “Severo Ochoa” (CSIC-UAM), Departamento de Biología Molecular, Universidad Autónoma de Madrid, Madrid, Spain; ^9^Department of Cell Biology, Physiology and Immunology, Maimonides Biomedical Research Institute of Cordoba (IMIBIC), University of Cordoba, Cordoba, Spain

**Keywords:** intravascular hemolysis, hemoglobin, heme, Nrf2, oxidative stress, tubular injury, sulforaphane

## Abstract

Massive intravascular hemolysis is associated with acute kidney injury (AKI). Nuclear factor erythroid-2-related factor 2 (Nrf2) plays a central role in the defense against oxidative stress by activating the expression of antioxidant proteins. We investigated the role of Nrf2 in intravascular hemolysis and whether Nrf2 activation protected against hemoglobin (Hb)/heme-mediated renal damage *in vivo* and *in vitro*. We observed renal Nrf2 activation in human hemolysis and in an experimental model of intravascular hemolysis promoted by phenylhydrazine intraperitoneal injection. In wild-type mice, Hb/heme released from intravascular hemolysis promoted AKI, resulting in decreased renal function, enhanced expression of tubular injury markers (KIM-1 and NGAL), oxidative and endoplasmic reticulum stress (ER), and cell death. These features were more severe in Nrf2-deficient mice, which showed decreased expression of Nrf2-related antioxidant enzymes, including heme oxygenase 1 (HO-1) and ferritin. Nrf2 activation with sulforaphane protected against Hb toxicity in mice and cultured tubular epithelial cells, ameliorating renal function and kidney injury and reducing cell stress and death. Nrf2 genotype or sulforaphane treatment did not influence the severity of hemolysis. In conclusion, our study identifies Nrf2 as a key molecule involved in protection against renal damage associated with hemolysis and opens novel therapeutic approaches to prevent renal damage in patients with severe hemolytic crisis. These findings provide new insights into novel aspects of Hb-mediated renal toxicity and may have important therapeutic implications for intravascular hemolysis-related diseases.

## Introduction

Recurrent and massive intravascular hemolysis induces renal toxicity, leading to acute kidney injury (AKI) and increasing the risk to develop chronic kidney disease (CKD) (Moreno et al., 2012; Naik et al., 2014). Intravascular hemolysis is a common characteristic of diseases such as sickle cell disease (SCD), paroxysmal nocturnal hemoglobinuria, hemolytic uremic syndrome, thrombotic thrombocytopenic purpura, and surgical procedures, including cardiopulmonary bypass or percutaneous mechanical thrombectomy (Ballarin et al., 2011; Baddam et al., 2017; Escobar et al., 2017; Mammen et al., 2017; Wetz et al., 2017; Guerrero-Hue et al., 2018).

Erythrocyte destruction leads to the release of hemoglobin (Hb) and heme-derived products to plasma, which are then filtered by kidney glomeruli and reabsorbed by the proximal tubular epithelium. Renal cell exposure to Hb and metabolites promotes side effects, such as inflammation, cell death, and oxidative stress characterized by lipid peroxidation and mitochondrial dysfunction (Balla et al., 1993; McFaul et al., 1994; Reiter et al., 2002; Liu and Spolarics 2003; Gonzalez-Michaca et al., 2004). Thus, in an oxidant milieu, heme-derived iron group may be oxidized, producing hydroxyl and hydroperoxyl radicals (Gutteridge, 1986). These radicals are highly reactive and promote the formation of reactive oxygen species and subsequent lipid peroxidation and DNA damage (Jia et al., 2007; Buehler et al., 2010). Endoplasmic reticulum (ER) stress, a common form of cell stress, has recently emerged as a pathophysiologic mechanism underlying the renal damage associated with Hb and heme accumulation (Deuel et al., 2016; Feng et al., 2018). ER stress is generated by the accumulation of misfolded proteins in the ER, initiating the unfolded protein response (UPR) that also can be triggered by several insults such as inhibition of proteasome, hypoxia, and oxidative stress (Kim et al., 2008; Liu et al., 2019). UPR compensates stress maintaining cellular homeostasis and avoiding cell death. Heme induces ER stress by expression of activating transcription factor-4 (ATF4) as well as splicing of X-box binding protein-1 (XBP1), promoting upregulation of targets such as CHOP (C/EBP homology protein) (Gall et al., 2018). Administration of transferrin, hemopexin, and haptoglobin resulted in beneficial extravascular/intravascular hemolytic anemias (Buehler and Karnaukhova, 2018). However, it is necessary to unravel the pathogenesis of Hb-mediated renal damage to identify novel therapeutic targets to protect against AKI and subsequent progression to CKD.

Nuclear factor erythroid-2-related factor 2 (Nrf2) is a transcription factor that drives the expression of target genes involved in cellular defense against xenobiotics and cellular stress such as oxidative and ER stress (Itoh et al., 1997; Cullinan and Diehl, 2004; Chang et al., 2018). In these harmful conditions, Nrf2 is translocated into the nucleus and interacts with antioxidant response elements (AREs) to upregulate a battery of phase II and antioxidant enzymes, such as HO-1 (the enzyme that catalyzes heme degradation) and ferritin (involved in iron storage and with ferroxidase activity) (Tsuji et al., 2000; Kobayashi and Yamamoto, 2006; Liu et al., 2009). Both proteins have been shown to be protective in heme-associated pathologies (Balla et al., 1992; Nath et al., 1992; Zarjou et al., 2013). Several studies have shown that Nrf2 induction protects against oxidative stress and inflammation in AKI (Shelton et al., 2013; Zarjou et al., 2013). Interestingly, pharmacological activation of Nrf2/HO-1 pathway was found to significantly protect against rhabdomyolysis-induced AKI (Shelton et al., 2013; Wang et al., 2014). Although Nrf2 activation has been postulated as a possible therapeutic target to treat diseases associated with intravascular hemolysis (Keleku-Lukwete et al., 2015; Doss et al., 2016; Ghosh et al., 2016; Vasconcellos et al., 2016; Belcher et al., 2017), additional studies are needed to unravel the specific effect of Nrf2 activation in kidney in this pathological setting. In the present study, we investigate the role of Nrf2 in Hb-induced AKI during intravascular hemolysis episodes in humans and in an experimental animal model resembling massive intravascular hemolysis. Nrf2 knockout mice showed more severe AKI. To further clarify the role of Nrf2 in AKI induced by intravascular hemolysis, we analyzed the expression of several Nrf2-related proteins. Finally, we also demonstrate that Nrf2 activation ameliorates AKI induced by intravascular hemolysis both *in vivo* and in cultured tubular epithelial cells, indicating that Nrf2 may be a therapeutic target for the treatment of these diseases.

## Material and Methods

### Human Renal Biopsy

We identified a renal biopsy from a 28-year-old patient with massive intravascular hemolysis secondary to percutaneous mechanical thrombectomy. At time of biopsy, the patient showed characteristics of AKI (sCr 9.78 mg/dl) and intravascular hemolysis (Hb 11 g/dl, platelets 180,000/µl, LDH 1,030 IU/L, and haptoglobin 5 mg/dl). Healthy kidney samples were obtained from non-tumor renal sections obtained after surgery in patients with kidney cancer and stored at the Instituto de Investigaciones Sanitarias-Fundacion Jimenez Diaz (IIS-FJD) biobank. Patients provided informed consent, and the biobank was approved by the IIS-FJD ethics committee.

### Animal Model

Intravascular hemolysis was induced by the intraperitoneal administration of a freshly prepared phenylhydrazine solution (2 mg/10 g of body weight) in 12-week-old wild-type C57BL/6 mice (Jackson Laboratory) or Nrf2-deficient mice (Nrf2−/−) (obtained from Dr. Susana Cadenas, CBMSO, Spain). Mice were housed in a pathogen-free, temperature-controlled environment with a 12-h/12-h light/dark photocycle and had free access to food and water. Phenylhydrazine hydrochloride (Sigma-Aldrich) was dissolved in phosphate-buffered saline (PBS) at a concentration of 10 mg/ml, and the pH was adjusted to pH 7.4 with NaOH. For Nrf2 activation, sulforaphane (12.5 mg/kg of body weight, Cayman Chemical) was administrated intraperitoneally 48, 24, and 2 h before phenylhydrazine injection. At 24 h after phenylhydrazine injection, mice were anesthetized (100 mg/kg of ketamine and 15 mg/kg of xylazine), saline perfused, and euthanized. Blood samples were collected for biochemistry analysis (ADVIA^®^ 2400 Clinical Chemistry System, Siemens Healthcare) and hematological analysis (Scil Vet ABC hematology analyser; Scil). Urine samples were collected for measuring urinary creatinine (creatinine assay kit, Abcam). The presence of heme in tissue, blood, and urine was quantified with a commercial kit (MAK316, Sigma). Dissected kidneys were fixed in 4% paraformaldehyde and embedded in paraffin for histological studies or snap frozen for RNA and protein studies, as previously described (Moreno et al., 2011; Sastre et al., 2013). All reported experiments were conducted in accordance with the Directive 2010/63/EU of the European Parliament and were approved by a local Institutional Animal Care and Use Committee (IIS-FJD).

### Immunohistochemistry/Immunofluorescence

Paraffin-embedded kidneys were cross-sectioned into 3-μm-thick pieces, and immunohistochemistry/immunofluorescence was performed as previously described (Rubio-Navarro et al., 2016). Specific primary antibodies were rabbit anti-Hb (1:100 dilution, ab92492, Abcam), rabbit anti-HO-1 (1:200 dilution, ADI-OSA-150-DEnzo Life technologies), rabbit anti-ferritin light chain (1:500 dilution, ab69090, Abcam), rabbit anti-phospho Nrf2 (1:50 dilution, bs-2013R, Bioss), Nrf2 (1:100 sc-722, Santa Cruz), rabbit anti-mouse 4-hydroxynonenal (4-HNE) (1:100, ab46545, Abcam), mouse anti-calnexin (1:100, 610523 BD Biosciences), and mouse anti-BiP (1:100, sc376768, Santa Cruz). The biotinylated secondary antibodies were applied for 1 h. Avidin–biotin peroxidase complex (Vectastain ABC kit, PK-7200, Vector Laboratories) was added for 30 min. Sections were stained with 3,3′-diaminobenzidine or 3-amino-9-ethyl carbazol (S1967, DAKO) and counterstained with hematoxylin. Images were taken with a Nikon Eclipse E400 microscope (Japan) and Nikon ACT-1 software (Japan). In immunofluorescence studies, slides were incubated with Alexa 488 as secondary antibodies (A11090, Invitrogen) and analyzed in an inverted confocal microscope (Leica TCS SP5). Nuclei were counterstained with 4′-6-diamidino-2-phenylindole (DAPI). Negative controls using the corresponding IgG were included to check for non-specific staining. Quantification of protein expression was performed using the Image-J software (National Institutes of Health, Bethesda, MD, USA) with similar acquisition settings in all tissues. Samples were examined in a blinded manner.

### TUNEL Assay

The degree of apoptosis was assessed using a terminal deoxynucleotidyl transferase (TdT) dUTP nick-end labeling (TUNEL) assay. Detection of DNA fragmentation was performed using a kit from Roche Applied Sciences (06432344001, Indianapolis, IN, USA). A semi-quantitative analysis was performed by counting the number of TUNEL-positive cells per field in the renal tissue at ×400 magnification. At least 10 areas in the cortex per slide were randomly selected. The mean number of green colored cells in these selected fields was expressed as the number of TUNEL-positive cells.

### Cell Culture

Proximal murine tubular epithelial (MCT) cells were cultured in RPMI 1640 (R0883, Sigma, MO, USA) supplemented with 10% decomplemented fetal bovine serum (FBS, F7524, Sigma, MO, USA), glutamine (2 mmol/L, G7513, Sigma, MO, USA), and penicillin/streptomycin (100 U/ml; P0781, Sigma, MO, USA) in 5% CO_2_ at 37°C. MCT cells were stimulated with Hb (0–500 µg/ml, corresponding to 0–30 µM of heme molar equivalents) (H0267, Sigma) and hemin/heme (0–30 µM) (H9039, Sigma). Some cells were pre-treated with sulforaphane (SFN, 2 μM, Cayman Chemical) or *tert*-butyl hydroquinone (tBHQ, 1 µM) (112941, Sigma) for 16 h before Hb/heme stimulation.

### Luciferase Assay in AREc32 Cell Cultures

Transformed MCF-7 breast cancer cells, stably expressing luciferase under the control of ARE sequences, AREc32 [kindly provided by Prof. Roland Wolf (University of Dundee, U.K.)], were cultured in DMEM with GlutaMAX and high glucose, supplemented with 1% penicillin-streptomycin (10,000 units), Geneticin (0.8 mg/ml), and 10% FBS at 37°C in a 5% CO_2_-supplemented air atmosphere. Nrf2 was induced in AREc32 cells as previously described (Buendia et al., 2015). In brief, AREc32 cells were seeded in 96-well white plates (2 × 10^4^ cells/well). Twenty-four hours later, cells were treated with Hb (0–500 µg/ml, 0–30 µM of heme molar equivalents) or heme (10 µM) for another 24 h. Thereafter, luciferase content was determined using a Luciferase Assay System (Promega E1500) and quantified in an Orion II microplate luminometer (Berthold, Germany).

### RNA Extraction and Real-Time PCR

Total RNA from kidneys or cultured cells was isolated with TriPure reagent (Roche) and reverse transcribed with High Capacity cDNA Archive Kit (Applied Biosystems). Real-time PCR was performed on ABI Prism 7500 PCR system (Applied Biosystems, Foster City, CA, USA) using the DeltaDelta Ct method.

Expression of target genes was analyzed by real-time quantitative PCR using Taqman^®^ gene expression assays for murine NGAL (Mm01324470_m1), KIM-1 (Mm00506686_m1), HO-1 (Mm00516005_m1), ferritin light chain (Mm03030144_g1), Nrf2 (Mm 00477784_m1), catalase (Mm00437992_m1), and NQO1 (Mm01253561_m1) (Applied Biosystems, Foster City, CA, USA) and designed probes for Atf4 (forward-5′-GGGTTCTGTCTTCCACTCCA-3′, reverse-5′-AAGCAGCAGAGTCAGGCTTTC-3′), CHOP/Ddit3 (forward-5′-CCACCACACCTGAAAGCAGAA-3′, reverse-5′-AGGTGAAAGGCAGGGACTCA-3′), spliced XBP1 (sXBP1) (forward-5′-CTGAGTCCGAATCAGGTGCAG-3′, reverse-5′-GTCCATGGGAAGATGTTCTGG-3′), ferritin heavy chain (forward-5′-AGACCGTGATGACTGGGAGA-3′, reverse-5′-TGAAGTCACATAAGTGGGGATCA-3′), and GAPDH (forward-5′-TGCACCACCAACTGCTTAGC-3′, reverse-5′-GGCATGGACTGTGGTCATGAG-3′) (Fisher Scientific, Spain). Expression levels are given as ratios to eukaryotic 18S rRNA (VIC, 4310893E).

### Western Blot

Tissue samples were homogenized in lysis buffer (50 mM of Tris–HCl, 150 mM of NaCl, 2 mM of EDTA, 2 mM of EGTA, 0.2% Triton X-100, 0.3% NP-40, 0.1 mM of PMSF, and 1 µg/ml of pepstatin A) and then separated by 10% SDS-PAGE under reducing conditions. After electrophoresis, samples were transferred to PVDF membranes (IPVH00010, Millipore, Bedford, MA, USA), blocked with 5% skimmed milk in TBS/0.5% v/v Tween 20 for 1 h, washed with TBS/Tween, and incubated with anti-HO-1 (1:2000 dilution, ADI-OSA-150-D Enzo Life Technologies), rabbit anti-ferritin light chain (1:500 dilution, ab69090, Abcam), rabbit anti-ferritin heavy chain (1:1000 dilution, Thermo Fisher 701934), and anti-phospho Nrf2 (1:1000 dilution, bs-2013R, Bioss). Antibodies were diluted in 5% milk TBS/Tween. Blots were washed with TBS/Tween and incubated with anti-rabbit horseradish peroxidase-conjugated secondary antibody (1:2000, Amersham, Aylesbury, UK). After being washed with TBS/Tween, blots were developed with the chemiluminescence method (ECL Luminata Crescendo, WBLUR0500, Millipore) and scanned using the ImageQuant LAS-4000 (GE Healthcare). Blots were then probed with mouse monoclonal anti-α-tubulin antibody (1:5000, T6199, Sigma, MO, USA), and levels of expression were corrected for minor differences in loading. Quantification was expressed as arbitrary densitometric units (AU).

### Assessment of Oxidative Stress

The molecular probe 2′,7′-dichlorodihydrofluorescein diacetate (H_2_DCFDA) (C6827; Invitrogen) was used to measure intracellular reactive oxygen species (ROS). Cells were incubated with H_2_DCFDA (5 µM) for 30 min, and ROS production was estimated using flow cytometry as previously reported (Rubio-Navarro et al., 2018). To determine cell superoxide anion production, cells were assayed with dihydroethidium (DHE) (Invitrogen) (Sastre et al., 2013). To quantify mitochondrial superoxide production, cells were incubated with MitoSOX Red (0.5 µM) for 30 min in the dark. Nuclei were counterstained with DAPI. Cells were then analyzed using confocal microscope (Leica TCS SP5).

### Cell Viability Assay

Cell viability was determined using the 3-[4,5-dimethylthiazol-2-yl]-2,5 diphenyltetrazolium bromide (MTT, Sigma) colorimetric assay. After treatment, cells were incubated with 1 mg/ml of MTT in PBS for 1 h at 37°C. The resulting formazan crystals were dried and dissolved in dimethylsulfoxide. Absorbance (indicative of cell viability) was measured at 570 nm in a microplate reader (BMG Labtech Offenburg, Germany).

### GSH Measurement

GSH quantification was performed as previously described (Kamencic et al., 2000). In brief, cells were incubated with monochlorobimane (100 µM) in free serum media for 45 min. Then, cells were washed twice with RPMI and treated for 6 h. Fluorescence intensity was measured in a Fluostar optima microplate reader (BMG Labtech Offenburg, Germany) at excitation and emission wavelengths of 410 and 485 nm, respectively.

### Statistical Analysis

Data were expressed as mean ± SEM. Differences between groups were analyzed with the Kruskal–Wallis test and the Mann–Whitney *U*-test. *p* values <0.05 were considered significant. Statistical analysis was performed using SPSS 11.0 statistical software.

## Results

### Nrf2 Is Activated in Human Kidney After Massive Intravascular Hemolysis

**Figure 1:**
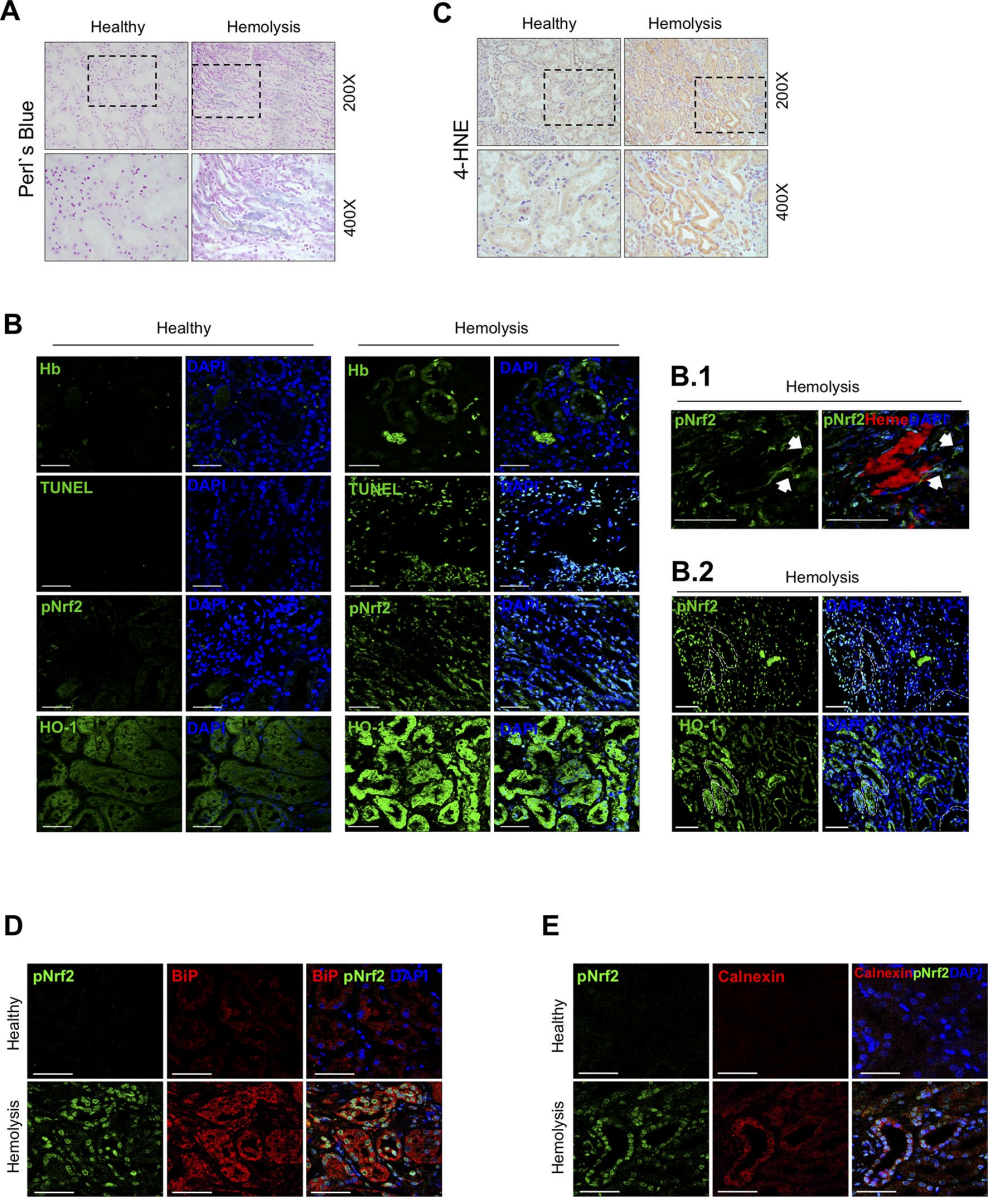
Nuclear factor erythroid-2-related factor 2 (Nrf2) activation in a patient with massive intravascular hemolysis-associated acute kidney injury (AKI). **(A)** Representative image of Perls’ Prussian blue staining showing iron accumulation in the renal biopsy of a patient with massive intravascular hemolysis as compared with healthy control (200× upper panel, 400× lower panel). **(B)** Representative confocal microscopy images showing Hb accumulation (green, first row), terminal deoxynucleotidyl transferase (TdT) dUTP nick-end labeling (TUNEL)-positive cells (green, second row), phospho-Nrf2 (green, third row), and HO-1 (green, fourth row). Nuclei were stained with 4′-6-diamidino-2-phenylindole (DAPI) (blue); scale bar, 100 µm. **B.1.** Representative confocal microscopy images showing the presence of heme cast (red) and nuclear phospho-Nrf2 (green); scale bar, 50 µm. **B.2.** Representative serial immunofluorescence images showing nuclear Nrf2 translocation (phospho-Nrf2, green) in HO-1-rich areas. White circles indicate similar regions in serial immunostained sections. Scale bar, 100 µm. **(C)** Representative image of 4-hydroxynonenal (4-HNE) staining (200× upper panel, 400× lower panel). Representative confocal microscopy images showing the presence of nuclear Nrf2 translocation (phospho-Nrf2, green) and the endoplasmic reticulum stress markers BiP **(**red, **D)** and calnexin **(**red, **E)**. Scale bar, 50 µm.

To determine whether Nrf2 is activated in kidneys as consequence of intravascular hemolysis, we performed histological studies in a patient with AKI-associated with intravascular hemolysis. Perls’ Prussian blue staining revealed iron accumulation in tubular cells compared with renal tissue from healthy donor (**Figure 1A**). Immunofluorescence studies confirmed the presence of Hb within tubular cells, as well as Nrf2 phosphorylation and translocation to nuclei and induction of HO-1 expression as compared with those of healthy control (**Figure 1B**). In this patient, we also observed increased tubular cell death, determined by TUNEL staining (**Figure 1B**), and exacerbated oxidative stress, as determined by 4-hydroxynonenal (4-HNE) staining (**Figure 1C**) and presence of the endoplasmic reticulum stress markers BiP (binding immunoglobulin protein) (**Figure 1D**) and calnexin (**Figure 1E**). These results suggest that kidney Hb/iron accumulation as a consequence of intravascular hemolysis promotes oxidative stress and tubular cell injury and further activation of Nrf2 in human disease.

### Hb/Heme Induces Nrf2 Activation in Both AREc32 Cells and Cultured Tubular Epithelial Cells

**Figure 2:**
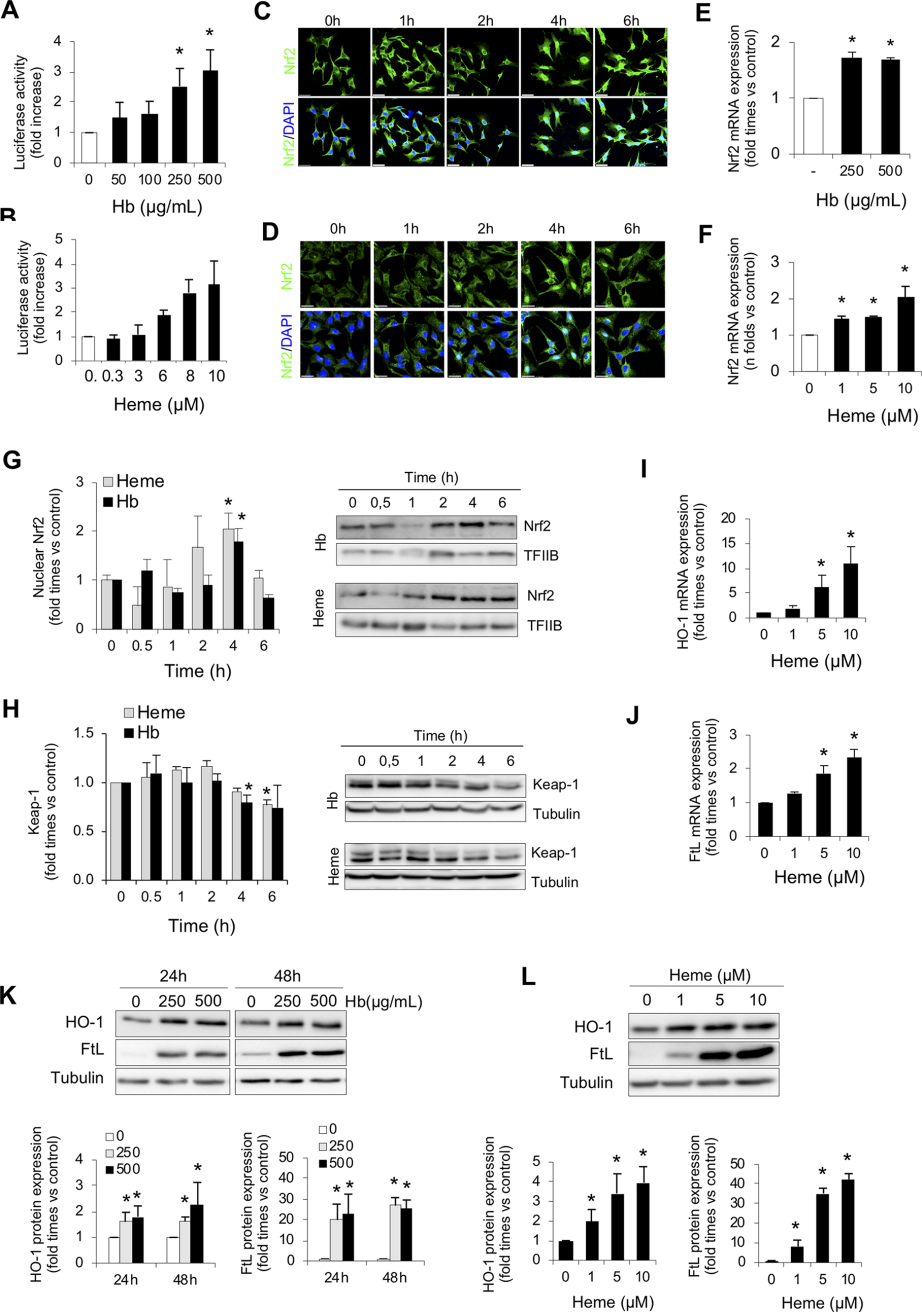
Hemoglobin and heme promote Nrf2 activation in cultured renal cells. Nrf2 transcriptional activity was measured by a luciferase reporter assay in AREc32 cells treated with increasing concentrations of Hb (0–500 µg/ml, 0–30 µM heme equivalents) **(A)** or heme (0–10 µM) **(B)** for 24 h. Representative confocal microscopy images showing Nrf2 nuclear translocation (green) in murine tubular (MCT) cells after exposure to Hb (500 µg/ml, 30 µM heme equivalents) **(C)** or heme (1 µM) **(D)** at different times (0–6 h). Nuclei were stained with DAPI (blue). Nrf2 mRNA expression measured by real-time-quatitative polymerase chain reaction (RT-qPCR) in MCT cells treated with Hb **(E)** or heme **(F)** for 6 h. **(G)** Nuclear Nrf2 levels in MCT cells treated with Hb (500 µg/ml, 30 µM heme equivalents) and heme (1 µM) at different times (0–6 h). Representative western blot image from nuclear protein fraction. TFIIB (transcription factor II B) was used as loading control. **(H)** Keap1 protein content in MCT cells treated with Hb (500 µg/ml, 30 µM heme equivalents) and heme (1 µM) at different times (0–6 h). HO-1 **(I)** and FtL **(J)** mRNA expression measured by RT-qPCR in MCT cells treated with heme for 6 h. **(K–L)** Representative western blot image showing HO-1 and FtL expression in MCT cells treated with Hb (0–500 µg/ml, 0–30 µM heme equivalents) and heme (0–10 µM) for up to 48 h (upper panel). Quantification of HO-1 and FtL by western blot (lower panel). Results are expressed as mean ± SE. *p < 0.05 vs non-treated cells.

To further explore how intravascular hemolysis modulates Nrf2 activity, we studied whether Hb and heme directly induce Nrf2 transcriptional activity in AREc32 cells. In these experiments, we observed that Hb or heme increased Nrf2-dependent luciferase reporter activity in a dose-dependent manner (**Figure 2A and B**). Moreover, we found that both Hb and heme induced Nrf2 mRNA expression and Nrf2 nuclear translocation and reduced the intracellular levels of the Nrf2 repressor Keap1 in a time-dependent manner in MCT (**Figure 2C–H**). Stimulation of MCT cells with Hb and heme increased mRNA and protein expression of two Nrf2-regulated proteins, HO-1 and ferritin subunits, heavy (FtH) (**Supplemental Figure 3A–C**) and light ferritin chain (FtL) (**Figure 2I–L**).These results suggest that intravascular hemolysis activates Nrf2 in the kidney *via* Hb and heme accumulation.

### Nrf2 Is a Protective Factor Against Kidney Injury Induced by Intravascular Hemolysis

**Figure 3:**
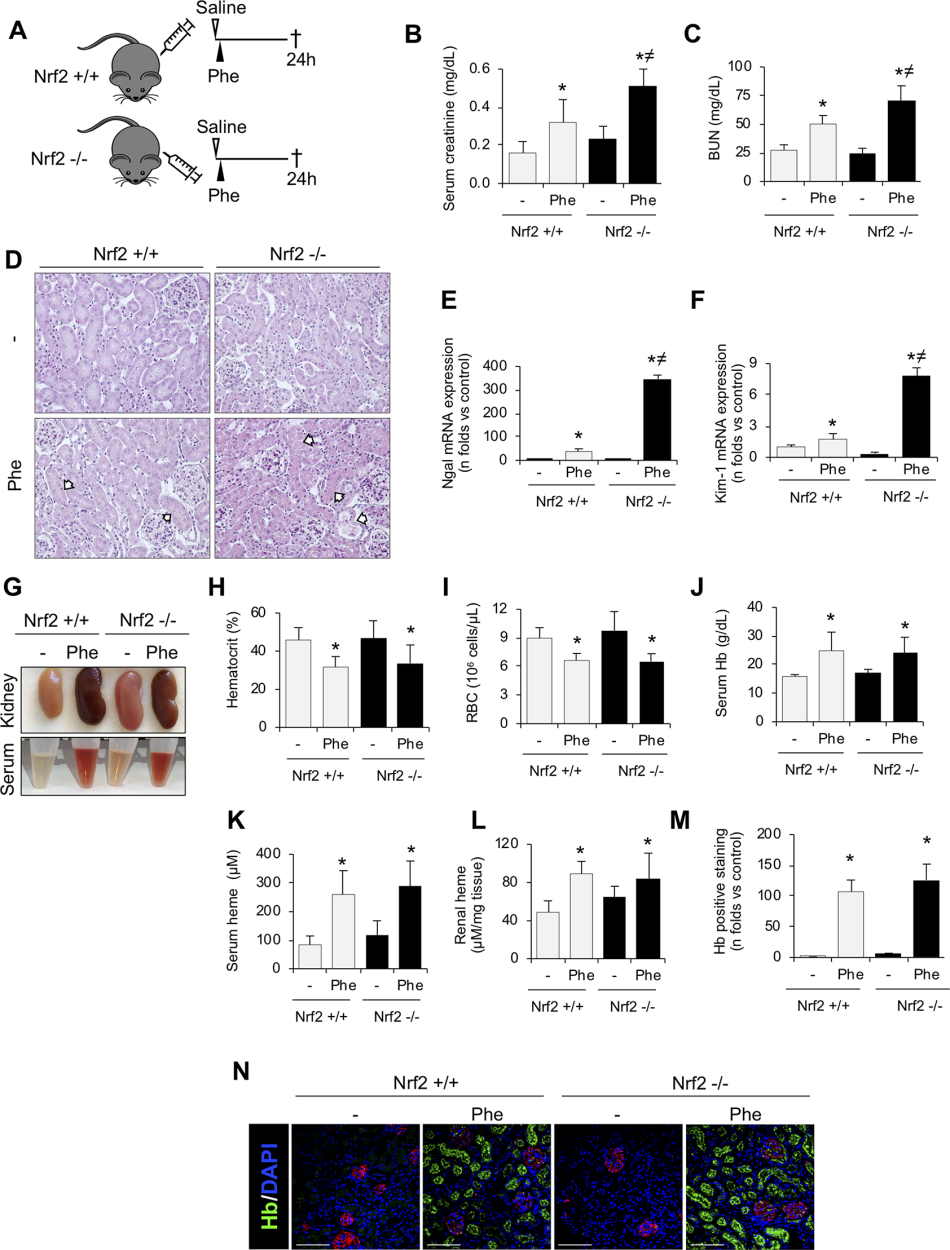
Nrf2 plays a protective role against kidney injury associated with intravascular hemolysis. C57BL/6 (Nrf2+/+) or Nrf2−/− mice (12 weeks old) were i.p. injected with saline (vehicle) or phenylhydrazine (Phe, 2 mg/10 g of body weight) to induce intravascular hemolysis (*n* = 8/group). **(A)** Schematic representation of intravascular hemolysis mouse model. Serum measurement of creatinine **(B)** and blood urea nitrogen (BUN) **(C)**. **(D)** Representative images showing hematoxylin and eosin staining in kidneys from mice with intravascular hemolysis. Arrows indicate signs of acute tubular injury: presence of intratubular debris, pyknotic nuclei from apoptotic cells, tubular epithelial cells into the lumen, and loss of nuclei in the tubular epithelium. Expression of tubular injury biomarkers NGAL **(E)** and KIM-1 **(F)**, as determined by real-time RT-qPCR, in kidneys from mice with intravascular hemolysis. **(G)** Representatives images showing kidneys (upper row) and serum (lower row) after intravascular hemolysis in both Nrf2+/+ and Nrf2−/− mice. Hematocrit **(H)**, total red blood cell (RBC) counts **(I)**, serum levels of Hb **(J)** and heme **(K)**, and heme concentrations in renal tissue **(L)**. **(M)** Semi-quantification of hemoglobin (Hb)-positive staining per renal cross section. **(N)** Representative images showing Hb (green) accumulation obtained by confocal microscopy. The podocyte marker nephrin (red) was used to delimitate the glomerular area. Nuclei were stained with DAPI (blue); scale bar, 100 µm. Results are expressed as mean ± SE. *p < 0.05 vs Nrf2+/+ control mice, ^≠^ *p* < 0.05 vs Nrf2+/+ Phe-injected mice.

To determine whether Nrf2 plays a protective role against hemolysis-induced kidney injury, we set up a mouse model of intravascular hemolysis induced by the intraperitoneal injection of phenylhydrazine in wild-type (Nrf2+/+) and Nrf2 knockout mice (Nrf2−/−) (**Figure 3A**). This is a well-established mouse model of AKI-associated with massive intravascular hemolysis promoted by phenylhydrazine-mediated lipid peroxidation of erythrocytes membranes, leading to the extracellular release of Hb and heme (Merle et al., 2018). In line with the human findings, we observed nuclear staining for phospho-Nrf2 (Ser40) (pNrf2) in kidneys from Nrf2+/+ mice with intravascular hemolysis (**Supplemental Figure 1**). Interestingly, our results show a decline of renal function (increase of serum creatinine and BUN levels) after induction of intravascular hemolysis in WT mice; and this was more severe in Nrf2−/− mice (**Figure 3B and C**). Similarly, we observed more severe histological changes (presence of intratubular debris, pyknotic nuclei from apoptotic cells, tubular epithelial cells into the lumen, and loss of nuclei in the tubular epithelium) and enhanced mRNA expression of the tubular injury biomarkers NGAL and KIM-1 in Nrf2−/− mice with intravascular hemolysis than in wild-type mice (**Figure 3D–F**). These data suggest that Nrf2 could be a protective factor against kidney injury promoted by intravascular hemolysis.

We next examined whether Nrf2 deficiency determines degree of systemic intravascular hemolysis. As expected, phenylhydrazine-injected mice showed a reduction in hematocrit and erythrocyte number, as well as an increased concentration of serum free Hb and heme, but no significant differences were observed between Nrf2+/+ and Nrf2−/− mice (**Figure 3G–K**). Hemolysis also promoted a similar renal accumulation of Hb and heme between mice strains, mainly in tubular cells (**Figure 3L–N**). Altogether, these data reveal that Nrf2 did not prevent erythrocyte lysis or Hb accumulation after the intravascular hemolysis episode.

### Nrf2 Protects From Kidney Oxidative Stress and Cell Death Promoted by Intravascular Hemolysis

**Figure 4:**
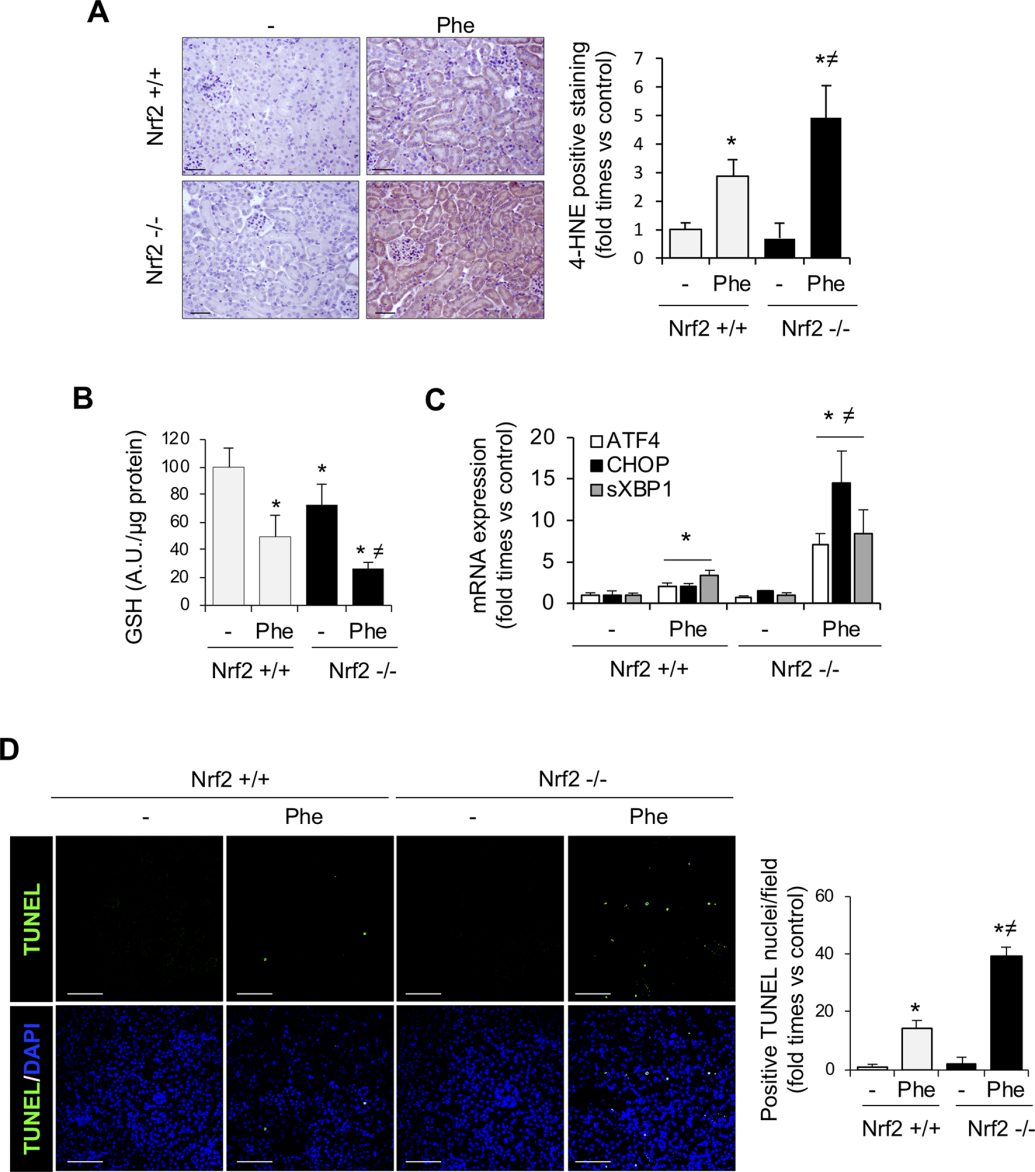
Nrf2 protects against oxidative stress and cell death associated with intravascular hemolysis. C57BL/6 (Nrf2+/+) or Nrf2−/− mice (12 weeks old) were i.p. injected with saline (vehicle) or phenylhydrazine (Phe, 2 mg/10 g of body weight) to induce intravascular hemolysis (*n* = 8/group). **(A)** Representative image of 4-hydroxynonenal (4-HNE) staining. Semi-quantification of 4-HNE-positive staining per renal cross section. **(B)** GSH content in renal tissue. **(C)** ATF4, CHOP, and sXBP1 mRNA levels determined in kidney by real-time RT-qPCR. **(D)** Representative confocal microscopy images showing nuclear staining of TUNEL (green) (left panel). Nuclei were stained with DAPI (blue); scale bar, 100 µm. Semi-quantitative analysis of TUNEL-positive cells (right panel). Results are expressed as mean ± SE. *p < 0.05 vs Nrf2+/+ control mice, ^≠^ *p* < 0.05 vs Nrf2+/+ Phe-injected mice.

To fully address the mechanism involved in Nrf2-mediated renal protection against intravascular hemolysis, we characterized oxidative stress and cell death, pathogenic processes characteristic of this scenario (Gutteridge, 1986; Gonzalez-Michaca et al., 2004). Intraperitoneal phenylhydrazine injection increased renal levels of 4-HNE, a marker of lipid peroxidation (**Figure 4A**), and decreased kidney GSH contents (**Figure 4B**). These effects were more severe in Nrf2−/− than in wild-type mice. Recently, ER stress has been shown as a new mechanism underlying the renal toxicity induced by Hb and heme accumulation (Deuel et al., 2016; Feng et al., 2018). We observed an increase in expression of UPR-related proteins such as CHOP, ATF4, and sXBP1 in kidney from WT mice injected with Phe. However, Nrf2−/− mice injected with phenylhydrazine showed a higher induction of these UPR-related genes (**Figure 4C**). Renal tubule cell death is an important consequence of Hb accumulation, leading to AKI and renal failure in patients with intravascular hemolysis (Guerrero-Hue et al., 2017). Therefore, we explored the potential beneficial effect of the Nrf2 transcription factor on cell death prevention. Intraperitoneal injection of phenylhydrazine induced tubular cell death, as determined by TUNEL-positive staining, in wild-type mice, an effect that was significantly increased in Nrf2−/− mice (**Figure 4D**).

**Figure 5:**
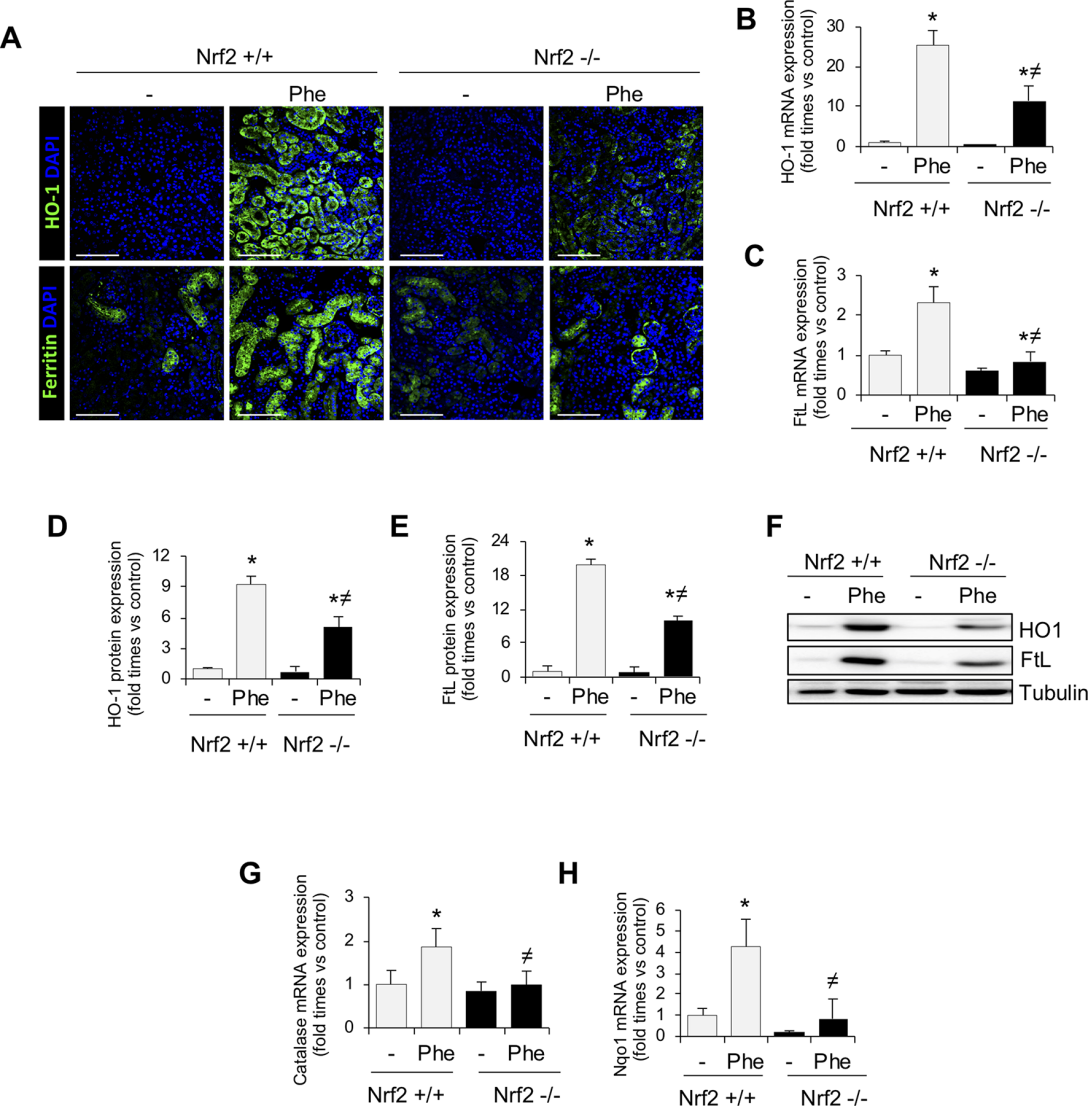
Nrf2-related proteins in kidney from mice with intravascular hemolysis. C57BL/6 (Nrf2+/+) or Nrf2−/− mice (12 weeks old) were i.p. injected with saline (vehicle) or phenylhydrazine (Phe, 2 mg/10 g of body weight) to induce intravascular hemolysis (*n* = 8/group). **(A)** Representative immunofluorescence images obtained by confocal microscopy showing expression of HO-1 (green, upper panel) and FtL (green, lower panel). Nuclei were stained with DAPI (blue); scale bar, 100 µm. HO-1 **(B)** and FtL **(C)** mRNA expression measured by RT-qPCR. Semi-quantification of HO-1 **(D)** and FtL **(E)** protein expression determined by western blot. **(F)** Representative western blot image of HO-1 amd FtL expression in kidneys from mice of the experimental model. Catalase **(G)** and NQO1 **(H)** mRNA expression measured by RT-qPCR. Results are expressed as mean ± SEM. *p < 0.05 vs Nrf2+/+ control mice, ^≠^ *p* < 0.05 vs Nrf2+/+ Phe-injected mice.

Next, to unravel the mechanism involved in Nrf2 protection, we analyzed the expression of some Nrf2-regulated genes, such as HO-1 and ferritin, which are implicated in the intracellular degradation of toxic heme (Stocker, 1990). Intravascular hemolysis upregulated HO-1 and both ferritin subunits, light ferritin (FtL) (**Figure 5A–F**) and heavy ferritin (FtH) (**Supplemental Figure 3D**). However, this compensatory response, consisting of heme-related and anti-oxidant proteins, was attenuated in Nrf2−/− as compared with wild-type mice, despite the more severe injury in Nrf2−/− mice. Similar effects were observed for other antioxidant Nrf2-dependent genes, such as catalase and NQO1 (**Figure 5G and H**). These results could explain why intravascular hemolysis induced more severe kidney injury in mice lacking Nrf2.

### Nrf2 Activation Protects Against Hb/Heme-Mediated Oxidative Stress and Cell Death *In Vitro*

**Figure 6:**
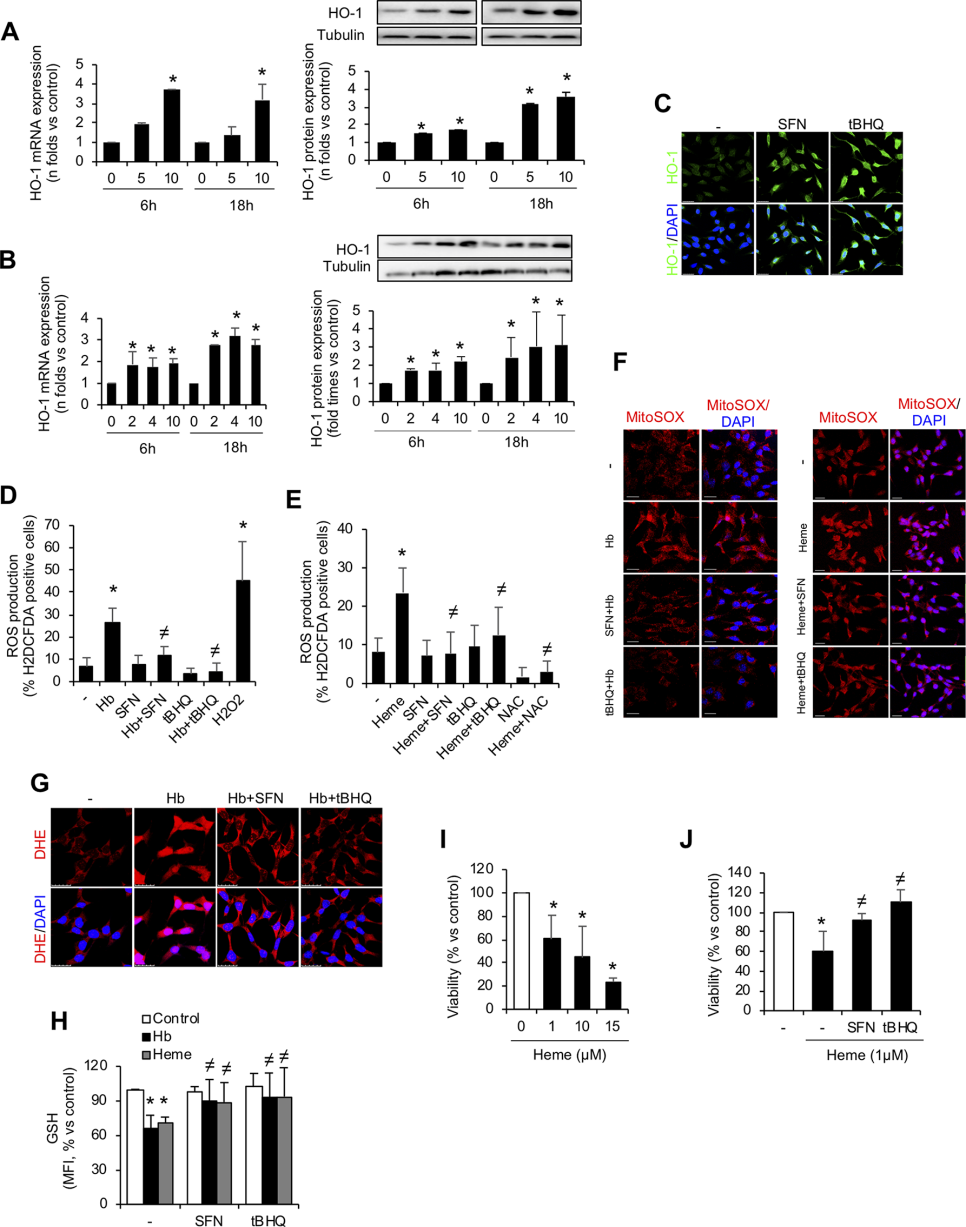
*In vitro* Nrf2 induction ameliorates oxidative stress and cell death. HO-1 mRNA and protein expression in MCT cells treated with the Nrf2 inducers *tert*-butylhydroquinone (tBHQ) **(A)** or sulforaphane (SFN) **(B)** at different concentrations (µM) for up to 18 h. **(C)** Representative confocal microscopy images showing HO-1 (green) staining in MCT cells pre-treated with Nrf2 inducers SFN and tBHQ with or without Hb. Nuclei were stained with DAPI (blue); scale bar, 20 µm. Quantification of ROS production (hydrogen peroxide) by flow cytometry with the fluorescent dye H_2_DCFDA in MCT cells stimulated with Hb **(D)** or heme **(E)** for 6 h. **(F)** Representative image of MitoSOX showing mitochondrial superoxide production by Hb (left panel) and heme (right panel) in cells pre-treated with SFN (2 µM) and tBHQ (1 µM) for 16 h. Nuclei were stained with DAPI (blue); scale bar, 20 µm. **(G)** Representative image of superoxide anion production determined by confocal microscopy using dihydroethidium (DHE) assay. Nuclei were stained with DAPI (blue); scale bar, 20 µm. **(H)** Intracellular GSH content in MCT cells treated with heme (1 µM) for 6 h with or without SFN (2 µM) or tBHQ (1 µM). **(I–J)** Cell viability determined in MCT cells stimulated with heme (1 µM) for 24 h and pre-treated with SFN (2 µM) or tBHQ (1 µM). Results are expressed as mean ± SEM. *p < 0.05 vs non-treated cells. ^≠^ *p* < 0.05 vs Hb or heme-treated cells.

We further investigated whether the Nrf2 inducers sulforaphane or *tert*-butylhydroquinone (tBHQ) protected MCT cells from Hb/heme-mediated damage. Thus, we performed *in vitro* experiments pre-treating MCT cells with sulforaphane or tBHQ for 16 h before adding Hb/heme. As expected, Nrf2 activators increased mRNA and protein HO-1 expression in time- and dose-dependent manner (**Figure 6A–C**). Importantly, we observed that sulforaphane and tBHQ reduced Hb- and heme-mediated ROS production, i.e., hydrogen peroxide (**Figure 6D and E**), mitochondrial superoxide (**Figure 6F**), and total superoxide (**Figure 6G**). Similarly, we found that pre-treatment with Nrf2 inducers abolished the Hb- and heme-mediated intracellular GSH reduction (**Figure 6H**). We next tested whether Nrf2 activation could protect tubular cells from cell death *in vitro*. Treatment with sulforaphane and tBHQ improved cell viability after exposure to heme (**Figure 6I and J**). Altogether, our results show that pharmacological activation of Nrf2 *in vitro* avoided Hb- and heme-mediated oxidative stress and cell death, indicating that Nrf2 could be a therapeutic target against kidney injury induced by intravascular hemolysis.

### *In Vivo* Nrf2 Activation Protects Against Intravascular Hemolysis-Associated AKI

**Figure 7:**
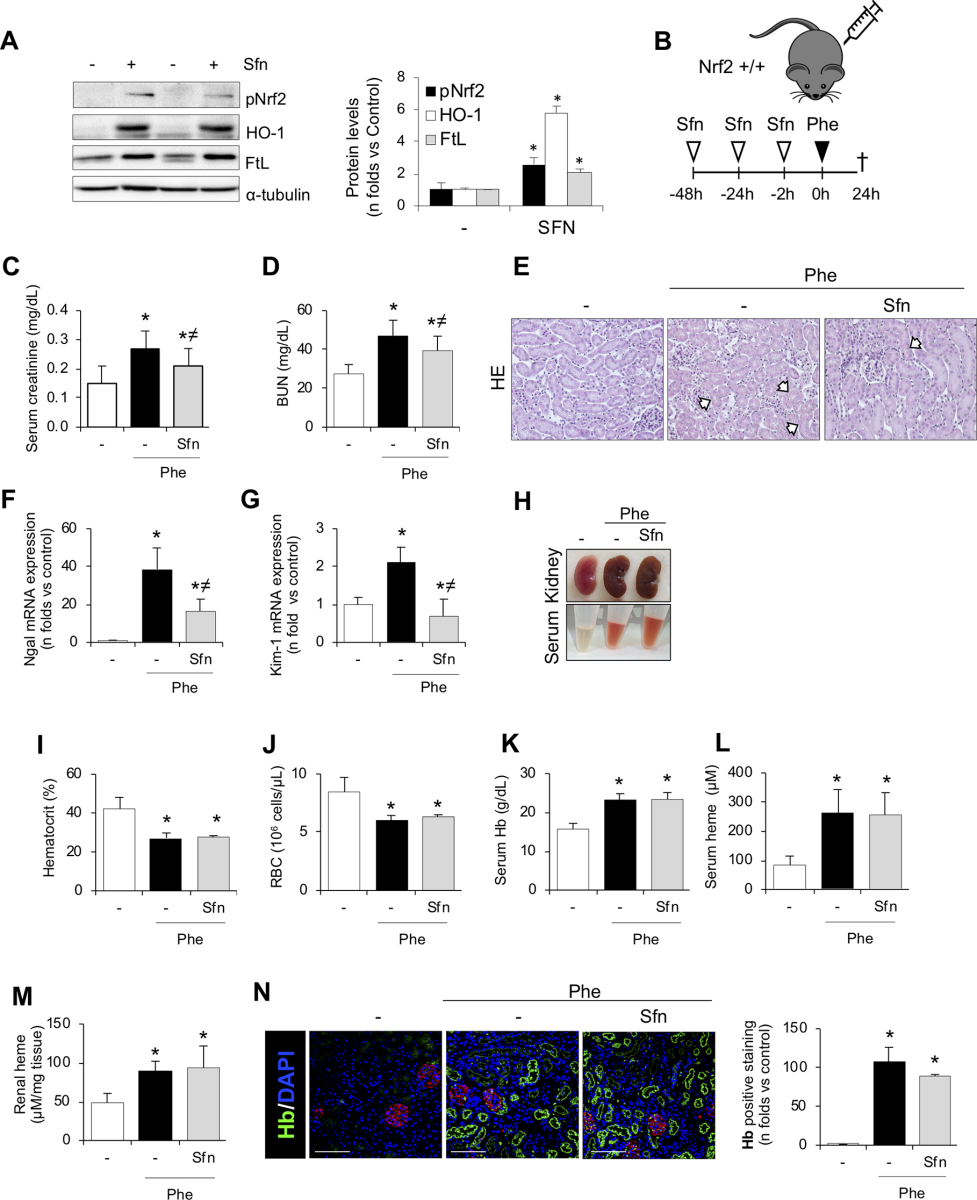
SFN treatment ameliorates acute kidney injury and tubular damage induced by intravascular hemolysis. **(A)** C57BI/6 mice (males, 12 weeks old) were i.p. treated with saline or sulforaphane (SFN, 12.5 mg/kg) for 3 days (*n* = 5/group). Representative western blot image showing phospho-Nrf2 (Ser40), HO-1, and FtL protein expression. **(B)** C57BI/6 mice (males, 12 weeks old) were i.p. treated with saline or sulforaphane (SFN, 12.5 mg/kg) for 48, 24, and 2 h before phenylhydrazine (*n* = 5/group). Mice were sacrificed 24 h after phenylhydrazine administration. Schematic representation of intravascular hemolysis mouse model. Serum measurement of creatinine **(C)** and blood urea nitrogen (BUN) **(D)**. Representative images showing hematoxylin and eosin staining **(E)**. Expression of tubular injury biomarkers NGAL **(F)** and KIM-1**(G)**, as determined by real-time RT-qPCR, in kidneys from mice with intravascular hemolysis. **(H)** Representatives images showing kidney and serum after intravascular hemolysis in mice treated with SFN. Hematocrit **(I)** and total red blood cell (RBC) counts **(J)**. Serum levels of Hb **(K)** and heme **(L)**. **(M)** Heme concentrations in renal tissue. **(N)** Representative images showing hemoglobin (Hb, green) accumulation obtained by confocal microscopy (left panel). The podocyte marker nephrin (red) was used to delimitate the glomerular area. Nuclei were stained with DAPI (blue); scale bar, 100 µm. Semi-quantification Hb-positive staining per cross section (right panel). Results are expressed as mean ± SEM. *p < 0.05 vs control mice, ^≠^ *p* < 0.05 vs Phe-injected mice.

Once we demonstrated the beneficial effects of Nrf2 activation *in vitro*, we tested whether this therapeutic approach may prove effective *in vivo*. Thus, in a first set of experiments, we administrated sulforaphane once a day for 3 days to analyze whether this treatment produced Nrf2 activation in kidney. As reported in **Figure 7A**, sulforaphane treatment induced Nrf2 phosphorylation and increased HO-1 and FtL levels in the kidney. We also ruled out any sulforaphane-derived adverse effect in the kidney (**Supplemental Figure 2**). We next conducted new experiments to determine whether sulforaphane administration protects from phenylhydrazine-mediated renal injury in mice (**Figure 7B**). Consistent with our previous findings, sulforaphane treatment improved renal function in mice with hemolysis, as assessed by serum creatinine and BUN (**Figure 7C and D**). Moreover, sulforaphane reduced histological injury (**Figure 7E**) and decreased the gene expression of the tubular injury markers KIM-1 and NGAL (**Figure 7F and G**). In line with our previous results, we did not observe any differences in the severity of hemolysis (hematocrit, erythrocyte number, and serum free Hb/heme concentration) between sulforaphane-treated and non-treated mice (**Figure 7H–L**). Moreover, kidney Hb/heme accumulation did not change according to sulforaphane treatment (**Figure 7M and N**).

**Figure 8:**
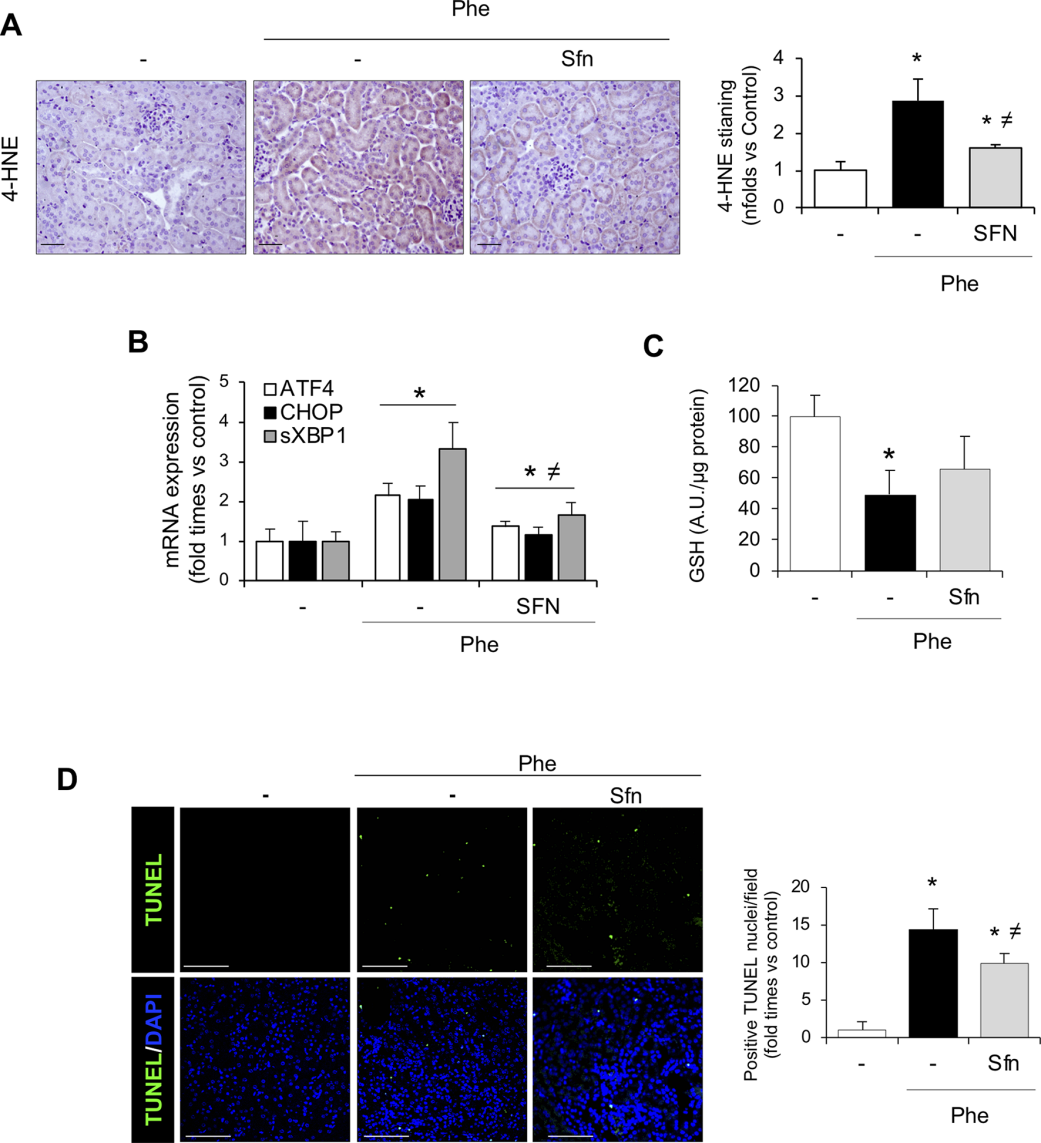
Nrf2 activation pathway in kidney from SFN-treated mice. **(A)** Representative image of 4-HNE staining in the experimental model (left). Semi-quantification 4-HNE-positive staining per cross section (right). **(B)** ATF4, CHOP, and sXBP1 mRNA levels determined in kidney by real-time RT-qPCR. **(C)** GSH content in renal tissue. **(D)** Representative confocal microscopy images showing nuclear staining of TUNEL (green) (left) and semi-quantitative analysis of TUNEL-positive cells (right). Nuclei were stained with DAPI (blue); scale bar, 100 µm. Results are expressed as mean ± SEM. *p < 0.05 vs control mice, ^≠^ *p* < 0.05 vs Phe-injected mice.

Interestingly, 4-HNE staining revealed lower oxidative stress in sulforaphane-treated than non-treated mice (**Figure 8A**). In addition, sulforaphane decreased the renal expression of UPR-related molecules induced by intravascular hemolysis (**Figure 8B**). Moreover, the decrease in renal GSH levels was milder in sulforaphane-treated mice with hemolysis and was not significantly different from that in controls as opposed to the significant GSH decrease observed in non-treated mice with hemolysis (**Figure 8C**). Finally, we observed that sulforaphane significantly decreased renal tubular epithelial cell death, evaluated by TUNEL staining, after intravascular hemolysis, as compared with that in non-treated mice (**Figure 8D and E**). Collectively, these results suggest that administration of the pharmacologic inducer of Nrf2, sulforaphane, relieves the kidney adverse effects observed in experimental hemolysis. We conclude that Nrf2 might be a potential therapeutic target for kidney injury induced by intravascular hemolysis.

## Discussion

In the present study, we demonstrate that Hb/heme accumulation in the kidney after massive intravascular hemolysis induces Nrf2 activation. We also found that massive intravascular hemolysis promotes AKI, resulting in increased serum creatinine concentration, tubular injury, oxidative and ER stress, and subsequent kidney cell death. These harmful effects were more severe in Nrf2 knockout mice, which showed a decreased expression of the Nrf2-dependent antioxidant proteins HO-1, FtL, and FtH and reduced levels of GSH, suggesting a critical protective role against Hb-mediated ROS production and cell toxicity in the kidney. Moreover, therapeutic Nrf2 activation reduced Hb/heme-mediated cellular stress and cytotoxicity in experimental hemolysis and cultured renal cells. Therefore, enhancing Nrf2 activity may be a potential therapeutic strategy to decrease renal injury associated with hemolytic diseases.

Recurrent or massive erythrocyte lysis increases plasma free Hb concentration and leads to renal Hb overload and toxicity, contributing to acute and chronic kidney injury (Tracz et al., 2007; Moreno et al., 2012; Naik et al., 2014). In our study, we administrated phenylhydrazine to induce severe hemolysis, thus promoting accumulation of Hb and its heme derivatives as well as a rapid decrease of renal function. It is well known that Hb triggers ROS production (Jia et al., 2007), similar to our observations in renal proximal tubular epithelial cells after exposure with Hb or heme. Recently, ER stress has been shown as a novel mechanism underlying the renal toxicity of Hb and heme (Deuel et al., 2016; Feng et al., 2018). Interestingly, Nrf2−/− mice showed an exacerbated renal expression of UPR-related genes, suggesting a protective role of this transcription factor against ER stress in kidney. Mitochondria are susceptible to Hb-mediated toxicity, resulting in decreased oxygen consumption and exacerbating mitochondrial ROS production (Nath et al., 1998). In this line, our results show an increased mitochondrial ROS production in renal cells exposed to Hb and heme. We also found that Hb/heme-mediated oxidative stress was related not only to production of superoxide anion and hydrogen peroxide but also to a reduction in GSH levels, the main cellular antioxidant. There is accumulating evidence supporting a direct link between mitochondria, oxidative stress, and cell death (Ott et al., 2007). Previous studies from our group showed that Hb induced apoptosis in renal podocytes by mitochondrial-related intrinsic pathway (Rubio-Navarro et al., 2018). Moreover, it has been shown that oxidative stress may contribute to ER stress and subsequent cell death (Liu et al., 2019). Specifically, rhabdomyolysis-induced ER stress promoted apoptosis, playing a crucial role in progression of AKI (Feng et al., 2018). According to these observations, the oxidative and ER stress as well as mitochondrial oxidation induced by Hb/heme may explain the increased cell death rate reported in our study, both *in vitro* and in mice with intravascular hemolysis (Nath et al., 1995; Gonzalez-Michaca et al., 2004). Moreover, this could explain the exacerbated cell death observed in kidney from Nrf2−/−, suggesting a protective role against renal toxicity subsequent to intravascular hemolysis.

There is no specific treatment to avoid the toxic effects caused by free Hb in the kidney. Therefore, understanding molecular mechanisms involved in this pathological setting is crucial to design new therapeutic strategies. In this line, Nrf2 has emerged as a master regulator of cellular resistance to oxidation (Guerrero-Hue et al., 2017). In basal conditions, Nrf2 is bound to Keap1, a cytosolic repressor that targets Nrf2 to the proteasome for degradation (Jaiswal, 2004). However, oxidative stress modifies cysteine residues in Keap1 and allows the nuclear translocation of Nrf2, such as what we observed in tubular cells stimulated with Hb and in mice with hemolysis. Once in the nucleus, Nrf2 transcriptionally induces the expression of cytoprotective enzymes and proteins involved in heme-degradation, including HO-1 and both ferritin subunits, FtH with ferroxidase activity and FtL that induces iron nucleation. Thus, we surmised that Nrf2 might improve intravascular hemolysis-induced kidney injury. To test this hypothesis, we induced intravascular hemolysis in both Nrf2+/+ and Nrf2−/− mice. Here, we show that mice lacking Nrf2 gene had more severe kidney injury, characterized by loss of renal function and increased cell stress and death. In agreement with our data, several studies have demonstrated the protective role of Nrf2 in a number of renal diseases associated with oxidative stress, such as cisplatin-induced nephropathy (Li et al., 2016), ischemia–reperfusion (Bayrak et al., 2008; Yoon et al., 2008; Trujillo et al., 2013), and rhabdomyolysis (Zhao et al., 2016). Oxidative stress drives ER stress and UPR activation in order to attenuate cellular stress through Nrf2 activation (Cullinan and Diehl, 2004; Liu et al., 2019). Interestingly, our data showed an increased expression of UPR-related proteins in kidneys from phenylhydrazine-injected Nrf2−/− mice as compared with wild-type mice, suggesting that the Nrf2 pathway may regulate kidney ER stress induced by intravascular hemolysis (Mukaigasa et al., 2018). In our study, the Nrf2-regulated molecules HO-1, FtL, and FtH were found upregulated in kidneys from wild-type mice after hemolysis or in cultured tubular epithelial cells stimulated with Hb/heme. Supporting the translational relevance of our findings, we observed increased Nrf2 and HO-1 expression, lipid peroxidation, and tubular cell death in a patient suffering from massive Hb renal accumulation after hemolysis-associated AKI. Similarly, patients with warm antibody hemolytic anemia, SCD, or thrombotic microangiopathy also showed increased expression and activity of HO-1 in renal tubules (Nath et al., 2001; Maroti et al., 2004; Fervenza et al., 2008). However, it is important to note that in our study, kidneys from Nrf2−/− mice with hemolysis showed an attenuated adaptive response characterized by lower expression of NQO-1, catalase, HO-1, FtH, and FtL than did wild-type mice with hemolysis. These Nrf2-regulated proteins play a key protective role against heme-mediated oxidative stress. HO-1 is the principal enzyme involved in heme degradation and, therefore, plays a cytoprotective function during heme-mediated tissue injury (Vogt et al., 1995). Interestingly, a known human case of HO-1 deficiency showed persistent hemolytic anemia with kidney iron deposits as well as loss of renal function (Yachie et al., 1999). Altogether, these data suggest the key role of the Nrf2/HO-1 axis in renal diseases associated with Hb accumulation. Recent studies have demonstrated the relevance of Nrf2 pathway in the regulation of hemolysis (Keleku-Lukwete et al., 2015; Krishnamoorthy et al., 2017). Thus, targeted disruption of Nrf2 induced hemolytic anemia in old mice by increasing erythrocyte susceptibility to oxidative stress (Lee et al., 2004). However, young Nrf2−/− mice did no present anemia (Chan et al., 1996). There are also conflicting reports on whether Nrf2 activation reduces hemolysis or not. Thus, the Nrf2 agonist dimethyl fumarate improved hematological parameters and reduced plasma-free Hb in SCD mice (Krishnamoorthy et al., 2017), whereas other authors showed that Nrf2 activation through ablation of its negative regulator Keap1 did not modify hemolysis and stress erythropoiesis in SCD mice, although it increased plasma heme clearance (Keleku-Lukwete et al., 2015). Our findings show no differences in serum free Hb and heme levels between Nrf2−/− and Nrf2+/+ mice or after Nrf2 activation with sulforaphane. Moreover, we do not observed differences in renal heme concentration according to Nrf2 genotypes or sulforaphane treatment. Altogether, these results seem to indicate that beneficial effects of Nrf2 on renal function are not related with a reduction in hemolysis or an increased heme catabolism in massive hemolysis induced by phenylhydrazine.

There is evidence that genetic or pharmacological Nrf2 activation may ameliorate systemic and vascular inflammation as well as lung injury and liver damage in mice with intravascular hemolysis (Keleku-Lukwete et al., 2015; Ghosh et al., 2016; Promsote et al., 2016; Belcher et al., 2017; Ghosh et al., 2018). However, none of these studies focused on renal injury, one of the major pathological consequences observed in patients with massive and recurrent hemolytic crises (Baddam et al., 2017; Mammen et al., 2017; Plewes et al., 2017). To investigate a potential beneficial effect of Nrf2 induction *in vivo*, we treated mice with sulforaphane before the onset of hemolysis. Remarkably, Nrf2 activation with sulforaphane ameliorated renal function and decreased oxidative and ER stress, cell viability, and tubular injury associated with massive hemolysis. Similar effects were observed in cultured tubular cells, where Nrf2 activation with sulforaphane or tBHQ reduced cellular and mitochondrial ROS production, the loss of GSH, and cell death induced by Hb and heme. These protective effects may be explained by an Nrf2-mediated induction of HO-1 and ferritin expression, thus decreasing heme-mediated toxic effects. In agreement with our data, Nrf2 activation also reduced cellular stress such as oxidative stress and ER stress and ameliorated renal function in experimental ischemia–reperfusion (Bayrak et al., 2008; Yoon et al., 2008; Trujillo et al., 2013) and rhabdomyolysis-associated AKI (Zhao et al., 2016; Guerrero-Hue et al., 2017). Interestingly, although activation of Nrf2 may induce UPR (Cullinan and Diehl, 2004; Liu et al., 2019), we did not observe significant changes in UPR-related genes in kidney from mice treated with sulforaphane compared with non-treated mice, suggesting that Nrf2 may activate UPR in the presence of ER stress but not in basal conditions. Moreover, different therapeutic approaches based on Nrf2 activation have preserved renal function in several past and ongoing clinical trials in patients with renal disease (Guerrero-Hue et al., 2017). Overall, our findings indicate for the first time that Nrf2 could be a potential target to prevent renal damage induced by intravascular hemolysis by ameliorating oxidative stress, ER stress, tubular cell death, and loss of renal function. These protective effects may be mediated almost in part by promoting the expression of antioxidant proteins involved in heme catabolism, including HO-1 and ferritin. Lack of cell-site-specific Nrf2 deletion in mice is a limitation of our study. Further studies must be performed to test whether specific Nrf2 deletion in tubular cells increases renal injury in hemolytic disorders.

In conclusion, our results show that Nrf2 plays a key regulatory role in limiting the severity of AKI triggered by intravascular hemolysis. Renal Hb accumulation promotes a loss of renal function and kidney injury, effects that are aggravated by the genetic deletion of Nrf2. Sulforaphane treatment induced Nrf2 activation as well as an increase of related proteins such as HO-1 and ferritin, decreasing the severity of kidney injury induced by intravascular hemolysis. Thus, Nrf2 could be a potential therapeutic target to limit Hb-induced toxicity in patients with intravascular hemolysis.

## Data Availability Statement

The raw data supporting the conclusions of this manuscript will be made available by the authors, without undue reservation, to any qualified researcher.

## Ethics Statement

Patients provided informed consent, and the biobank was approved by Instituto de Investigaciones Sanitarias-Fundacion Jimenez Diaz (IIS-FJD) ethics committee. All reported experiments with animals were conducted in accordance with the Directive 2010/63/EU of the European Parliament and were approved by Instituto de Investigaciones Sanitarias-Fundacion Jimenez Diaz Animal Care and Use Committee.

## Author Contributions

AR-N and JM designed the study. AS, CY, and ER were involved in human samples collection. PC analyzed renal biopsies. AR-N, CV-C, CH, RL, PM, MG-H, SC, MG, JE, IC, CG-C, and BA performed research. AR-N, CV-C, MG-H, and JM performed data analysis and data interpretation. EG, AR-N, JE, AO, MP, and JM wrote the manuscript. All authors critically revised the manuscript for important intellectual content.

## Funding

Supported by FIS/FEDER CP14/00008, CP16/00014, CP16/00017, PI15/00448, PI16/00735, PI16/02057, PI17/00130, PI17/01495, PI17/01700, ISCIII-RETIC REDinREN RD012/0021, RD016/0009 FEDER funds, Spanish Ministry of Economy and Competitiveness (RYC-2017-22369), Sociedad Española de Nefrología, Fundacion Renal Iñigo Álvarez de Toledo (FRIAT), Comunidad de Madrid CIFRA2 B2017/BMD-3686 and BMD-3827, Fundacion La Caixa, CaixaImpulse program CI17-00048, and Spanish Biomedical Research Centre in Diabetes and Associated Metabolic Disorders (CIBERDEM).

## Conflict of Interest Statement

The authors declare that the research was conducted in the absence of any commercial or financial relationships that could be construed as a potential conflict of interest.

## Supplementary Material

Figure S1 Nrf2 resulted activated in mice with intravascular hemolysis. Representative confocal microscopy images showing nuclear translocation of phosphorylated-Nrf2 (Ser40) (green) in C57Bl/6 wild type (Nrf2+/+) mice that were i.p. injected with saline (Vehicle) or phenylhydrazine (Phe, 2mg/10g of body weight) to induce intravascular hemolysis. Nuclei were stained with DAPI (blue), scale bar 100 µm.

Figure S2 Expression of NGAL **(A)** and KIM-1 **(B)** determined by RT-qPCR in kidneys from control mice treated with sulforaphane (SFN, 12.5 mg/kg) for 3 days. These animals did not display evidence of tubular injury as there was no increase in the expression of these markers. **(C)** Quantification of total Nrf2 protein in kidney from control mice and treated with SFN (left panel). Representative western blot image showing total Nrf2 protein levels (right panel). Results are expressed as mean ± SE. *p<0.05 vs non-treated cells.

Figure S3
**(A)** Expression of FtH mRNA expression measured by RT-qPCR in MCTs cells treated with heme for 6h. **(B)** Western blot image showing FtH expression in MCT cells treated with Heme (0-10 µM) for 24h. **(C)** FtH protein expression in MCT cells stimulated with Hb (0-500 µg/mL, 0-30 µM heme equivalents). FtH mRNA expression measured by RT-qPCR **(D)** and semiquantification of FtH protein expression determined by western-blot **(E)** of kidneys from wild type and Nrf2 -/- mice injected with phenylhydrazine or vehicle. FtH mRNA expression measured by RT-qPCR **(F)** and semiquantification of FtH protein expression determined by western-blot **(G)** of kidneys from wild type pre-treated with SFN and injected with phenylhydrazine or vehicle.
