# Supplementary figures and images for: Nrf2 Plays a Protective Role Against Intravascular Hemolysis-Mediated Acute Kidney Injury
Source: Front Pharmacol. 2019 Jul 3;10:740. doi: 10.3389/fphar.2019.00740 (PMC6619398; doi:10.3389/fphar.2019.00740)

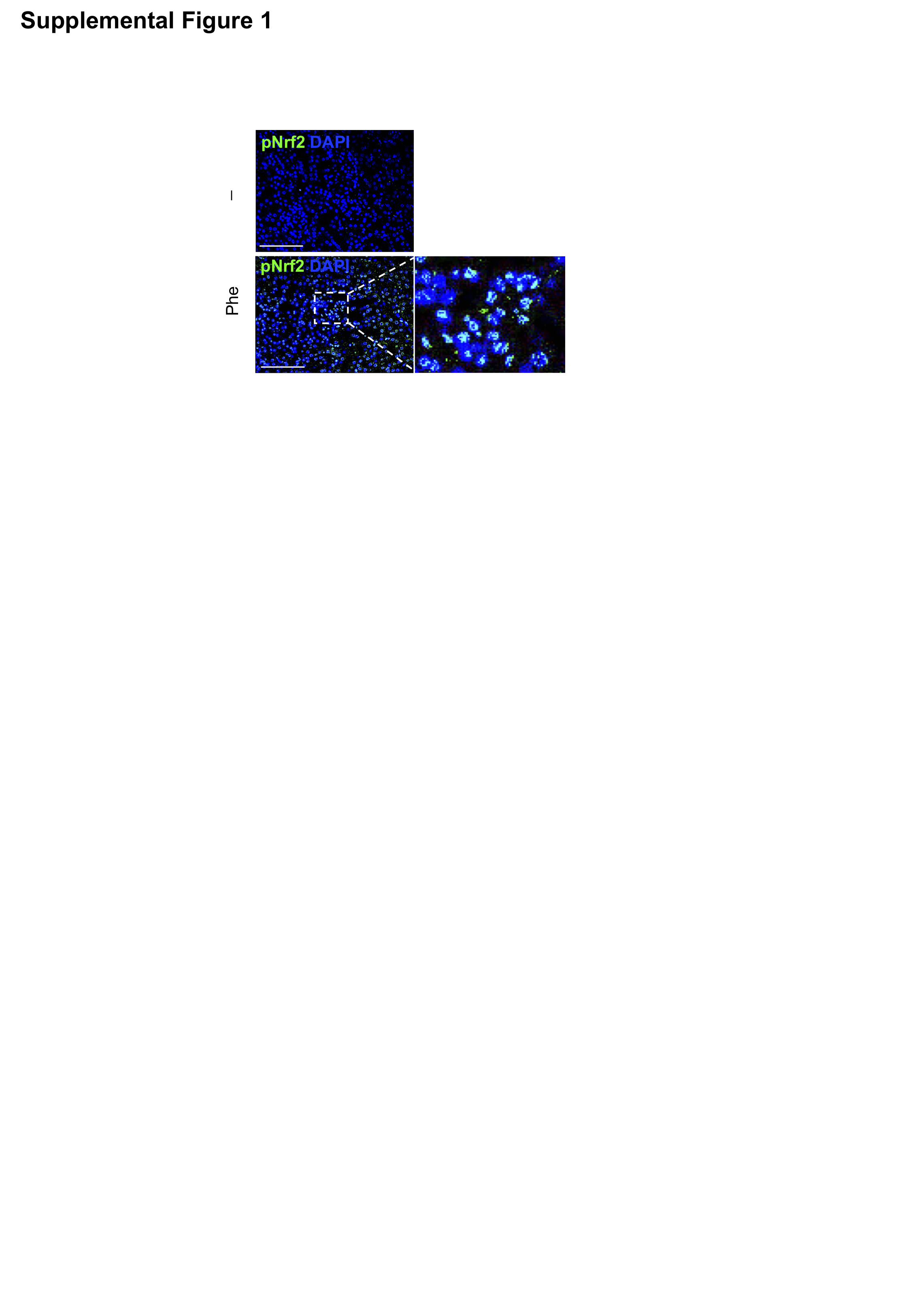

Supplement: Figure S1 — Nrf2 resulted activated in mice with intravascular hemolysis. Representative confocal microscopy images showing nuclear translocation of phosphorylated-Nrf2 (Ser40) (green) in C57Bl/6 wild type (Nrf2+/+) mice that were i.p. injected with saline (Vehicle) or phenylhydrazine (Phe, 2mg/10g of body weight) to induce intravascular hemolysis. Nuclei were stained with DAPI (blue), scale bar 100 µm. [file Image_1.tiff]

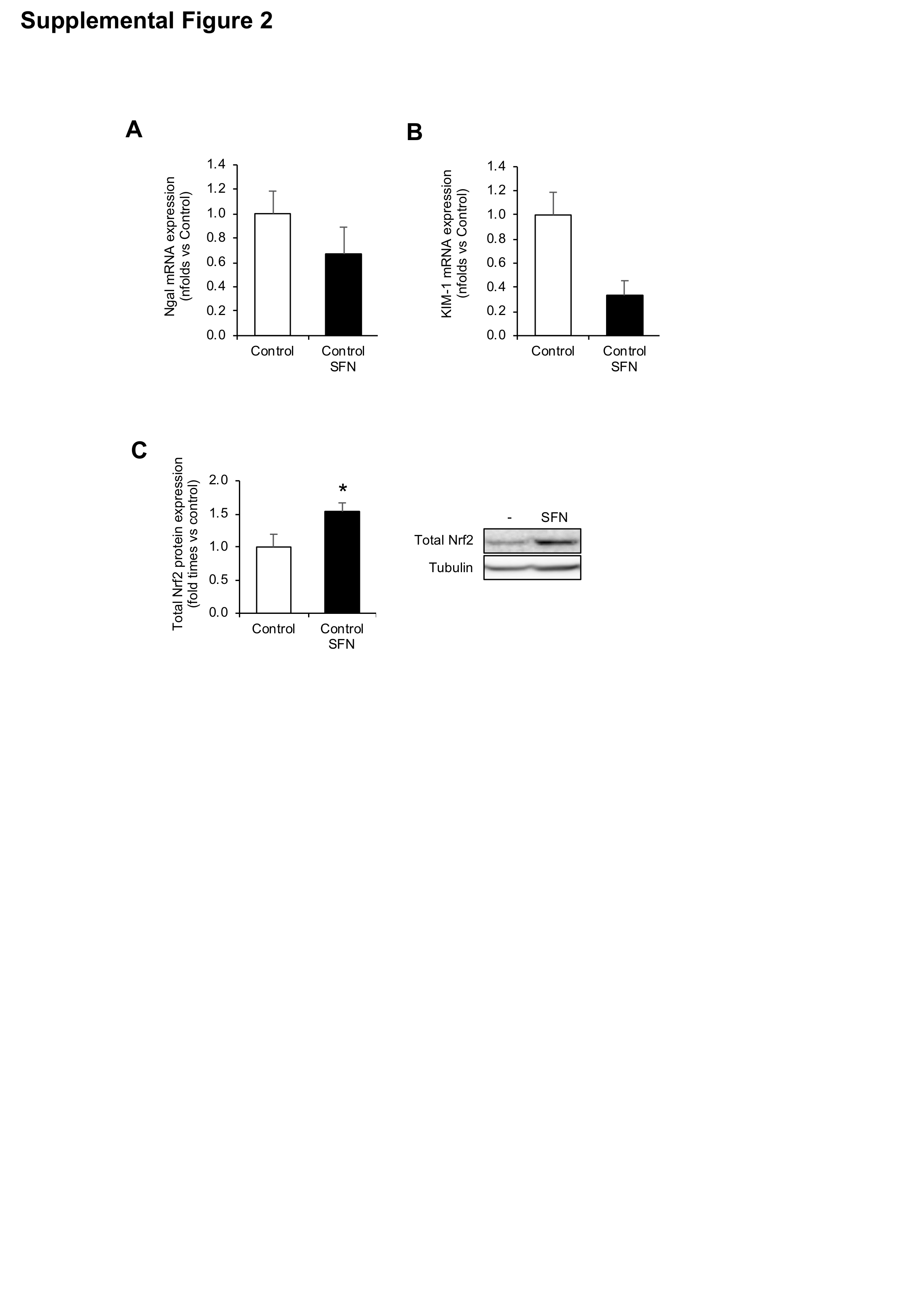

Supplement: Figure S2 — Expression of NGAL (A) and KIM-1 (B) determined by RT-qPCR in kidneys from control mice treated with sulforaphane (SFN, 12.5 mg/kg) for 3 days. These animals did not display evidence of tubular injury as there was no increase in the expression of these markers. (C) Quantification of total Nrf2 protein in kidney from control mice and treated with SFN (left panel). Representative western blot image showing total Nrf2 protein levels (right panel). Results are expressed as mean ± SE. *p<0.05 vs non-treated cells. [file Image_2.tiff]

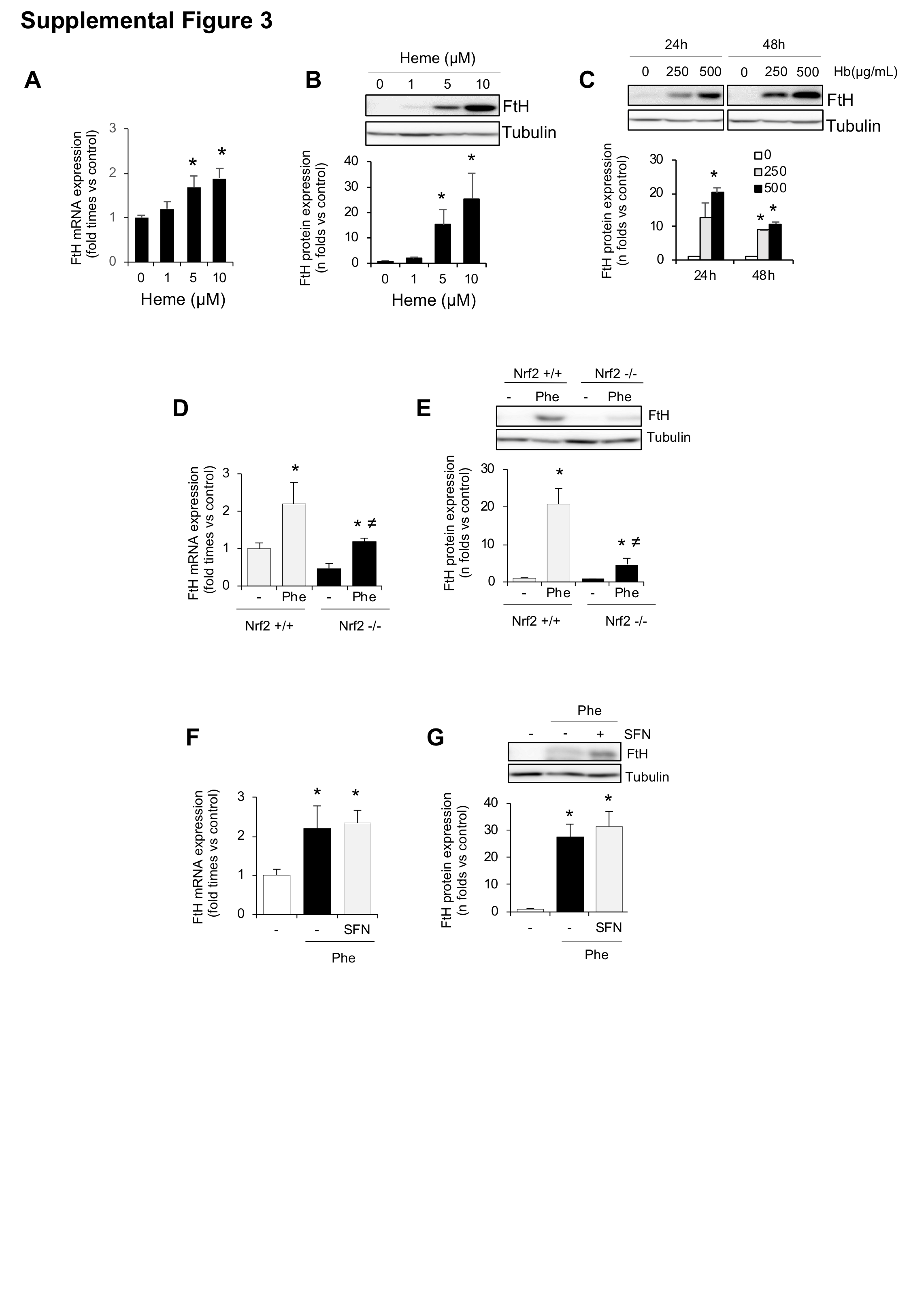

Supplement: Figure S3 — (A) Expression of FtH mRNA expression measured by RT-qPCR in MCTs cells treated with heme for 6h. (B) Western blot image showing FtH expression in MCT cells treated with Heme (0-10 µM) for 24h. (C) FtH protein expression in MCT cells stimulated with Hb (0-500 µg/mL, 0-30 µM heme equivalents). FtH mRNA expression measured by RT-qPCR (D) and semiquantification of FtH protein expression determined by western-blot (E) of kidneys from wild type and Nrf2 -/- mice injected with phenylhydrazine or vehicle. FtH mRNA expression measured by RT-qPCR (F) and semiquantification of FtH protein expression determined by western-blot (G) of kidneys from wild type pre-treated with SFN and injected with phenylhydrazine or vehicle. [file Image_3.tiff]
